# Supplementary material for: Comparative Transcriptome Analyses Provide Potential Insights into the Molecular Mechanisms of Astaxanthin in the Protection against Alcoholic Liver Disease in Mice
Source: Mar Drugs. 2019 Mar 19;17(3):181. doi: 10.3390/md17030181 (PMC6471478; doi:10.3390/md17030181)
Supplement: Supplementary file 1 [file marinedrugs-17-00181-s001.pdf]

Supplementary File

Table S1. Statistics of RNA-seq.

Sample	Reads Num.	Bases (bp)	Q20 (%)	Q30 (%)	GC(%)
AST1	57306828	8596024200	96.94	92.73	48.69
AST2	58171262	8725689300	96.95	92.74	48.91
AST3	51068172	7660225800	97.15	93.17	47.78
AST4	54090536	8113580400	97.08	93.03	48.74
Con2	53850852	8077627800	96.86	92.57	49.35
Con3	51597436	7739615400	96.96	92.76	49.02
Con4	50463722	7569558300	97.06	92.97	48.37
Con5	52238762	7835814300	97.03	92.9	49.07
Et1	59958048	8993707200	97.13	93.08	49.52
Et2	54805428	8220814200	97.25	93.33	49.35
Et3	54682914	8202437100	97.14	93.14	48.76
Et4	63385554	9507833100	97.24	93.31	49.29
EtAST1	53333880	8000082000	97.3	93.43	48.24
EtAST2	75319414	1.1298E+10	96.63	92.11	48.56
EtAST3	75499284	1.1325E+10	97.03	92.88	47.87
EtAST4	74737096	1.1211E+10	97.35	93.56	48.02

Table S2. Sequences of primers used for real-time RT-PCR analyses

Gene Name	5′primer	3′primer
18s	CTCAACACGGGAAACCTCAC	CGCTCCACCAACTAAGAACG
IL-6	TGGAAATGAGAAAAGAGTTGTGC	CCAGTTTGGTAGCATCCATCA
TNF-a	GGTGGTTTGCTACGACGTG	TTCATCTTTGAAGAAGAGCCCAT
IL-1a	CAAGCAACGGGAAGATTCTG	CTGATCTGGGTTGGATGGTC
IL-1b	TTCATCTTTGAAGAAGAGCCCAT	TCGGAGCCTGTAGTGCAGTT
MCP-1	GGCTCAGCCAGATGCAGT	TGAGCTTGGTGACAAAAACTACAG
MIP-2	GCGCCCAGACAGAAGTCATA	TCCAGGTCAGTTAGCCTTGC
TLR2	GCAAACGCTGTTCTGCTCAG	AGGCGTCTCCCTCTATTGTATT
TLR3	GTGAGATACAACGTAGCTGACTG	TCCTGCATCCAAGATAGCAAGT
TLR4	ATGGCATGGCTTACACCACC	GAGGCCAATTTTGTCTCCACA
TLR6	TGAGCCAAGACAGAAAACCCA	GGGACATGAGTAAGGTTCCTGTT
NLRP3	ATCAACAGGCGAGACCTCTG	GGGACATGAGTAAGGTTCCTGTT
NLRP1a	GGTGGTGTGAAGATGTTGTGT	TCCATGTTCATCGTAGGGACC
IL-18	GACTCTTGCGTCAACTTCAAGG	CAGGCTGTCTTTTGTCAACGA
Caspase-1	AATACAACCACTCGTACACGTC	AGCTCCAACCCTCGGAGAAA
MyD88	TCATGTTCTCCATACCCTTGGT	AAACTGCGAGTGGGGTCAG
LCN2	ATGTCACCTCCATCCTGGTC	ACCTGAGGATACCTGTGCAT

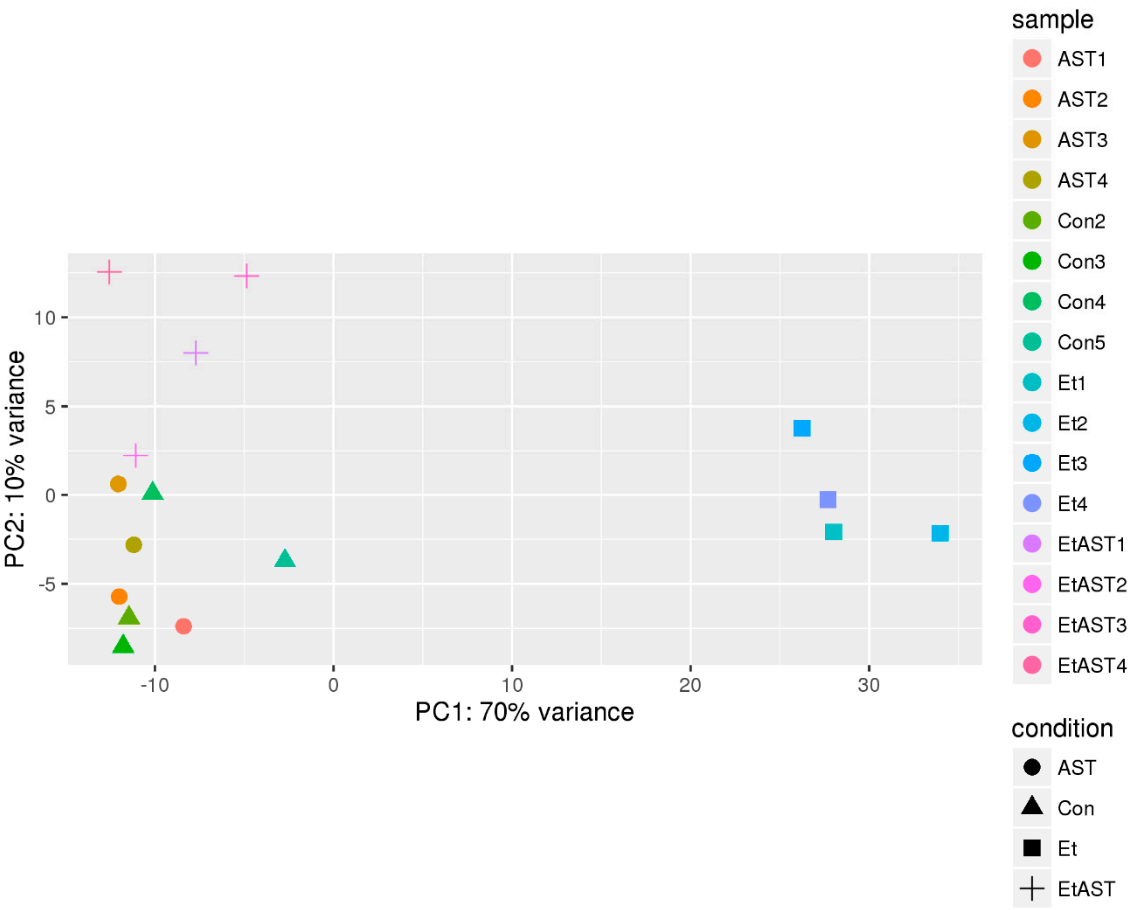

Figure S1. Principal Components Analysis. x-axis represents the first principal component, y-axis represents the second principal component; different shapes represent different groups; different colors represent the different samples.

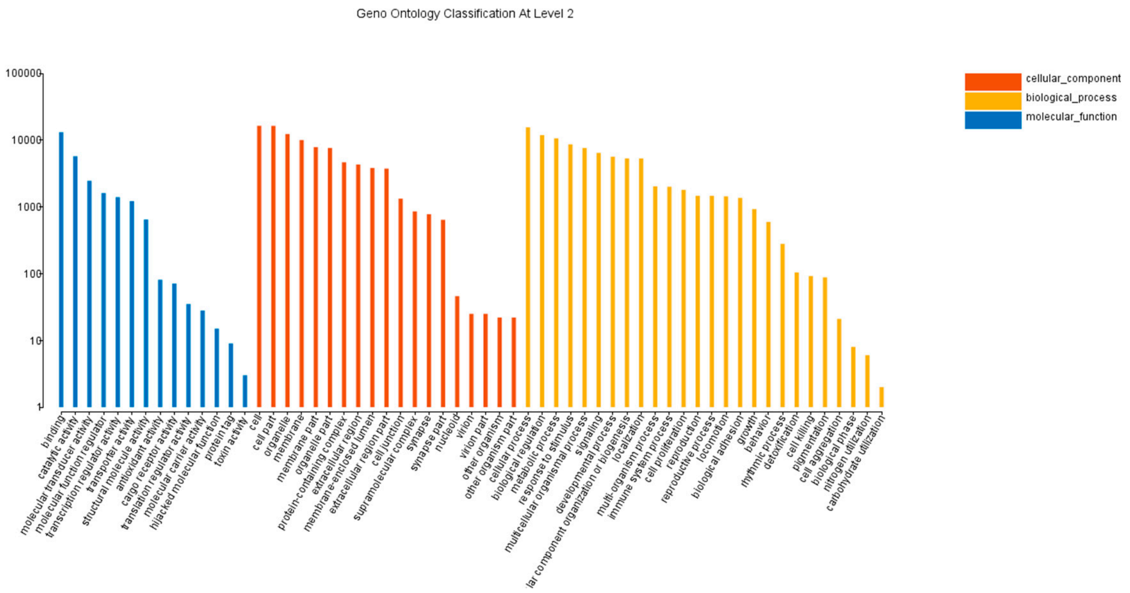

Figure S2. Histogram presentation of gene distribution in Gene Ontology (GO) functional classification. The x-axis represents level to GO terms; the left y-axis represents gene numbers in each GO term. Genes were further classified into sub-groups in biological process, cellular component, and molecular function.

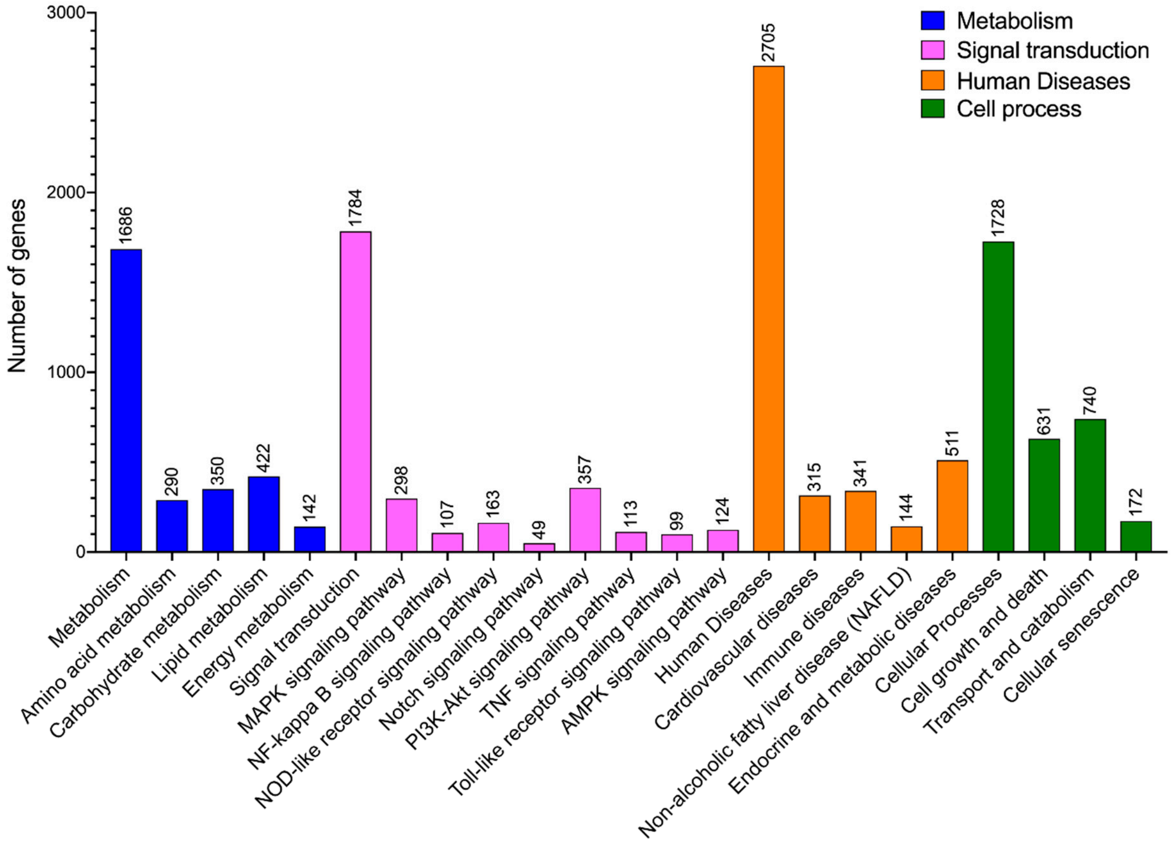

Figure S3. Histogram presentation of gene distribution in KEGG classification. The x-axis represents level to KEGG terms; the left y-axis represents gene numbers in each term. Genes were further classified into sub-groups in metabolism, signal transduction, human diseases, and cell process.

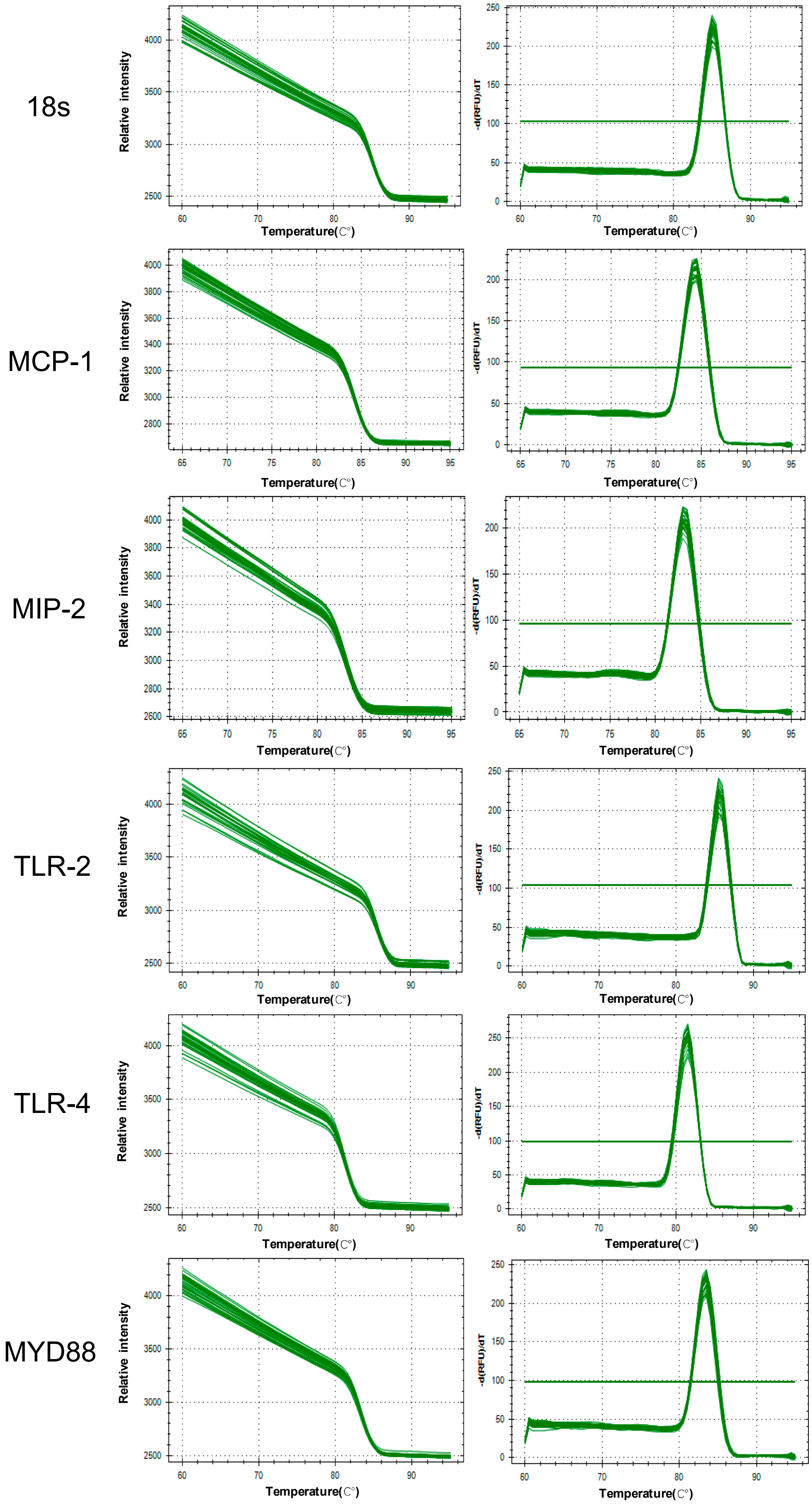
18s
MCP-1
MIP-2
TLR-2
TLR-4
MYD88
Relative intensity
-d(RFU)/dT
Temperature(C°)

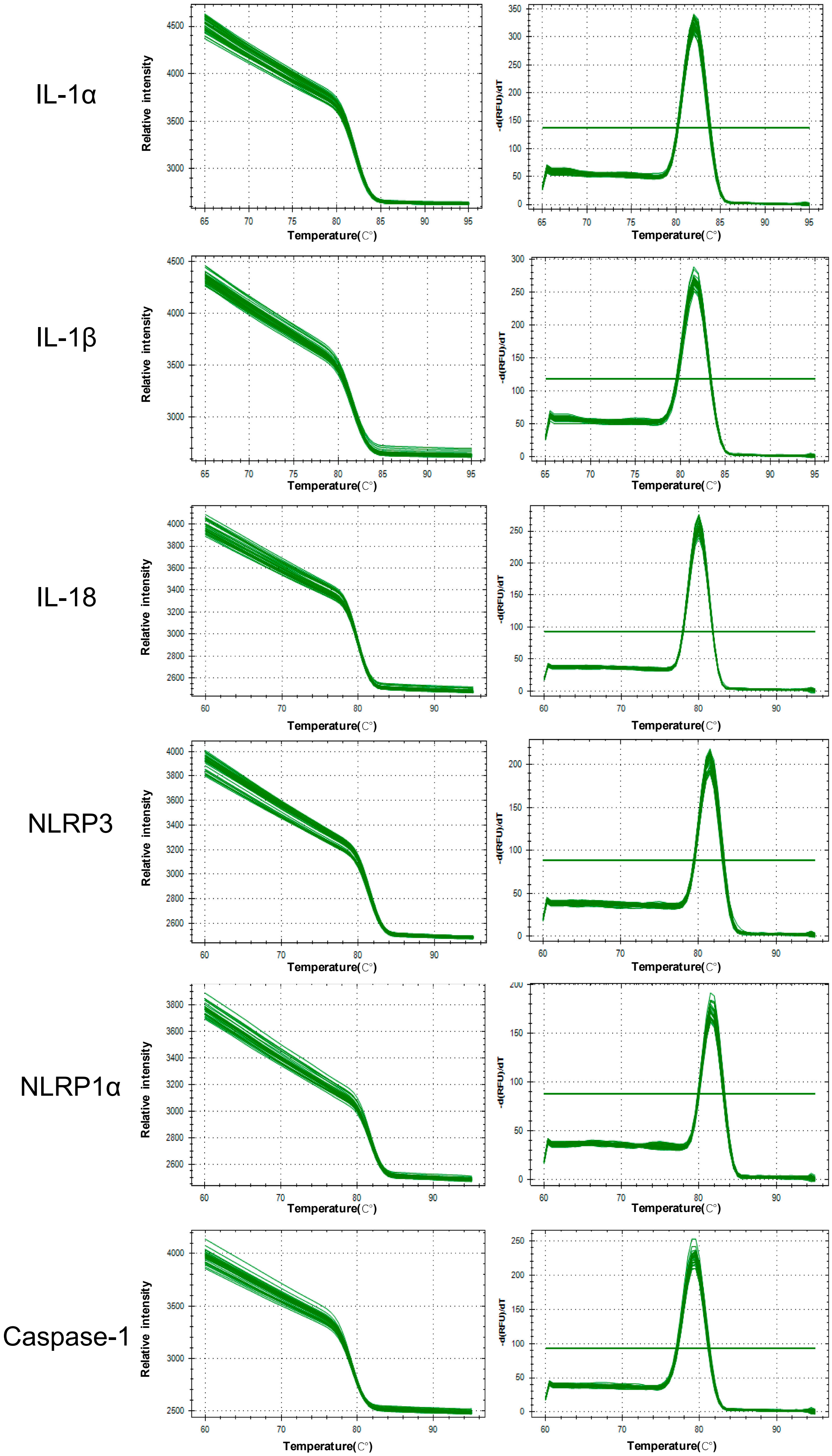
IL-1α
IL-1β
IL-18
NLRP3
NLRP1α
Caspase-1
Relative intensity
-d(RFU)/dT
Temperature(C°)

Figure S4. Determination of qPCR melting curves and melting peaks for selected genes involved in NOD-like pathway, Toll-like pathway, and chemokines pathway. Left side: qPCR melting curves, the x-axis represents melting temperature; the left y-axis represents relative fluorescence intensity. Right side: qPCR melting peaks, the x-axis represents melting temperature and the y-axis is the relative fluorescence unit (RFU) rate of change over time (T) (-d(RFU)/dT).

© 2018 by the authors. Submitted for possible open access publication under the terms and conditions of the Creative Commons Attribution (CC BY) license (http://creativecommons.org/licenses/by/4.0/).
